# Combined aqupla, paclitaxel liposome, and docetaxel treatment: survival and biomarker outcomes in recurrent ovarian cancer patients

**DOI:** 10.3389/fonc.2024.1422117

**Published:** 2024-06-17

**Authors:** Jie Yang, Mengyu Zhang, Yilei Zhang, Lanfen Zhu, Qiming Wang

**Affiliations:** Department of Gynecology, Women’s and Children’s Hospital of Ningbo University, Ningbo, Zhejiang, China

**Keywords:** aqupla, paclitaxel liposome, docetaxel, recurrent ovarian cancer, survival, biomarkers

## Abstract

As one lethal malignancy in women’s reproductive systems, ovarian cancer (OC) is frequently detected at an advanced phase during diagnosis. when the disease has spread widely. The absence of obvious symptoms and powerful screening tools in the early stages makes treatment difficult and the prognosis poor. Despite the clinical remission that can be achieved in some patients after initial treatment, the recurrence rate is conspicuous, posing a considerable challenge in treating recurrent OC (ROC). In the retrospective analysis, we compared the effects of two treatment regimens, aqupla combined with paclitaxel liposome (NP group) versus aqupla combined with docetaxel (ND group), on survival and biomarkers in patients with ROC. The study included 121 OC patients, and clinical data were collected through an electronic medical record system, outpatient review records, and a follow-up record system. The results revealed a notably higher overall remission rate in the ND group than the NP group, but revealed no notable inter-group discrepancy in toxicities, implying that the aqupla combined with docetaxel regimen may be more effective in platinum-sensitive ROC patients. Additionally, post-treatment CA125 levels were lower in patients in the ND group, suggesting that the regimen may be more effective in reducing tumour load. Survival analysis further revealed that treatment regimen, FIGO stage, number of recurrent lesions, and pretreatment CA125 level were independent prognostic factors affecting patients’ 5-year OS and PFS. Overall for ROC patients, especially platinum-sensitive patients, the aqupla in combination with docetaxel regimen provided an improved survival benefit with a comparable safety profile, highlighting the importance of individualised treatment strategies.

## 1 Introduction

Ovarian cancer (OC) is one prevalent malignancy among gynaecological tumours, but also one of the most lethal (1). OC usually occurs in hard-to-detect areas, the early signs are atypical and variable, and there is a lack of efficient early detection methods (2). As a result, more than 70 per cent of patients have advanced cancer when diagnosed (3). For patients with advanced disease, whose long-term survival is not promising, tumour cytoreduction is the most common treatment option, supplemented by a combination of platinum and paclitaxel chemotherapy after surgery (4). Unfortunately, about 70–80 per cent of patients with moderate to advanced disease experience disease recurrence (5, 6). In the management of relapses, there is no consistent pattern of treatment, and chemotherapy is at the centre of treatment.

However, not all patients have the opportunity to undergo secondary tumour cytoreduction. For some patients, the risks and burdens of a second surgery may be beyond their reach for a variety of personal reasons, including family and financial constraints, as well as medical considerations (7, 8). This means that these patients can only choose chemotherapy as their primary treatment. Platinum-based chemotherapy is the mainstay in treating recurrent OC (ROC) and involves a large number of patients. In preparing a treatment plan for these patients, due consideration needs to be given to the possible side effects and complications arising from the different treatment options, as well as to the impact on the survival of the patients (9) Aqupla is a second-generation platinum-based chemotherapeutic agent. Compared with cisplatin and carboplatin, aqupla reduces nephrotoxicity and gastrointestinal side effects to a certain extent while maintaining similar anti-tumour activity (10). Inhibits the proliferation and growth of cancer cells through forming crosslinks with DNA and blocking DNA replication and transcription. Paclitaxel liposome is a liposomal formulation of paclitaxel which, by encapsulating paclitaxel in liposomes, improves its pharmacokinetic properties and increases its concentration in tumour tissues while reducing its toxicity to normal tissues (11). Docetaxel is another microtubule stabiliser with a mechanism similar to paclitaxel but with a different chemical structure (12). Docetaxel inhibits cell division by preventing the depolymerisation of microtubules, leading to cancer cell death (13). The combination of aqupla with paclitaxel liposome and docetaxel is common in treating ROC, but the advantages and disadvantages of the two regimens remain controversial.

In this study, a comparative analysis of the effects of two therapeutic regimens, aqupla combined with paclitaxel liposome and aqupla and combined with cetaxel, was conducted in ROC, aiming to investigate the differences between the two regimens in terms of therapeutic effect, patient survival and The study is to investigate the discrepancies between these two regimens regarding therapeutic efficacy, patient survival, and adverse effects, as well as the primary factors impacting the survival of ROC patients.

## 2 Materials and methods

### 2.1 Clinical data collection

Retrospective analysis of clinical data of OC patients treated at our hospital from Jan. 2015 to Jan. 2019. The research was conducted with permission of Women’s and Children’s Hospital of Ningbo University Medical Ethics Committee (Approval No. EC2024–030).

The research is based on the electronic medical record system, outpatient review record and follow-up record system to obtain the relevant information of patients. Clinical information included: International Federation of Gynecology and Obstetrics Staging (FIGO) staging (14), age, Pathology Type, Initial Surgical Treatment, Eastern Cooperative Oncology Group Performance Status (ECOG) Score (15), Maximum diameter of recurrent lesions, number of recurrent lesions, response to platinum drugs, history of diabetes mellitus, history of hypertension, clinical outcome of the patient, incidence of adverse events. Laboratory parameters include: pre- and post-treatment Carcinoembryonic Antigen (CEA), Cancer Antigen 19–9 (CA19–9), Cancer Antigen 125 (CA125), Cluster of Differentiation 4 (CD4), Cluster of Differentiation 3 (CD3), as well as Cluster of Differentiation 8 (CD8).

### 2.2 Inclusion and exclusion criteria

Inclusion criteria: (1) confirmed diagnosis of OC, fallopian tube cancer, primary peritoneal cancer after pathological examination; (2) recurrence after achieving complete remission level in primary treatment, first recurrence, platinum-free interval >6 months; (3) tumour markers and imaging tests suggesting recurrence, no contraindication to surgery or chemotherapy; (4) complete case data; (5) follow up to the survival outcome.

Exclusion criteria: (1) previous history of other malignant tumours; (2) history of severe allergy to platinum and other drugs; (3) bone marrow dysfunction. (4) Combined liver and kidney function abnormalities. (5) Undergoing secondary tumour cytoreduction after recurrence. (6) Expected survival time of the patient is less than 6 months.

### 2.3 Patient grouping

Based on the inclusion and exclusion criteria, we obtained 121 eligible cases. A query of the electronic medical record system revealed that 64 patients received aqupla combined with paclitaxel liposome treatment (NP group), while 57 patients received aqupla combined with docetaxel treatment (ND group).

### 2.4 Treatment regimen

NP group: On the first day, patients were treated with paclitaxel liposome (product code H20030357, 30 mg, manufacturer: Nanjing Green Leaf Pharmaceutical Co., Ltd.) at a dose of 130–170 mg/m², mixed with 500 ml of dextrose solution (5%) and administered through intravenous drip over 3 h. On the second day, aqupla (Product No. H20143133, 20 mg, Manufacturer: Jiangsu Oseikang Pharmaceutical Co., Ltd.) was used at 85–105 mg/m², mixed with 500 ml of 0.9% saline, by intravenous drip over 1 hour. After completion of the drip, the intravenous drip was continued with 1500 ml to 2000 ml of 0.9% saline.

ND group: On the first day, patients were treated with docetaxel (Product No. H20093092, 20 mg, Manufacturer: Zhejiang Haizheng Pharmaceutical Co., Ltd.) at 60–100 mg/m², mixed with 500 ml of dextrose solution (5%), and used intravenously for more than 30 min. The second day of aqupla treatment was comparable to the NP group.

Both groups will be treated in three-week cycles. At each cycle, the patient’s systemic status and treatment efficacy will be assessed. If the patient deteriorates or cannot tolerate the treatment, the chemotherapy regimen will be discontinued. Patients in both groups will receive two to six cycles of chemotherapy.

### 2.5 Follow-up

The follow-up duration in the study was five years. We used medical record searches as well as outpatient and telephone visits to follow up the patients. During the five-year duration, we kept detailed records of the patients’ outpatient visits. We also looked at the patient’s overall health status, including but not limited to whether the disease had recurred or metastasised, as well as whether the patient had survived or died. Five-year over survival (OS): This is calculated from the time the patient was first diagnosed with a relapse until the patient’s death within five years. Progression-Free Survival (PFS): The duration from the end of the patient’s first relapse therapy to the point of disease progression, relapse, or patient death, with the first of these three conditions as the endpoint.

### 2.6 Clinical outcome assessment

**Figure 1:**
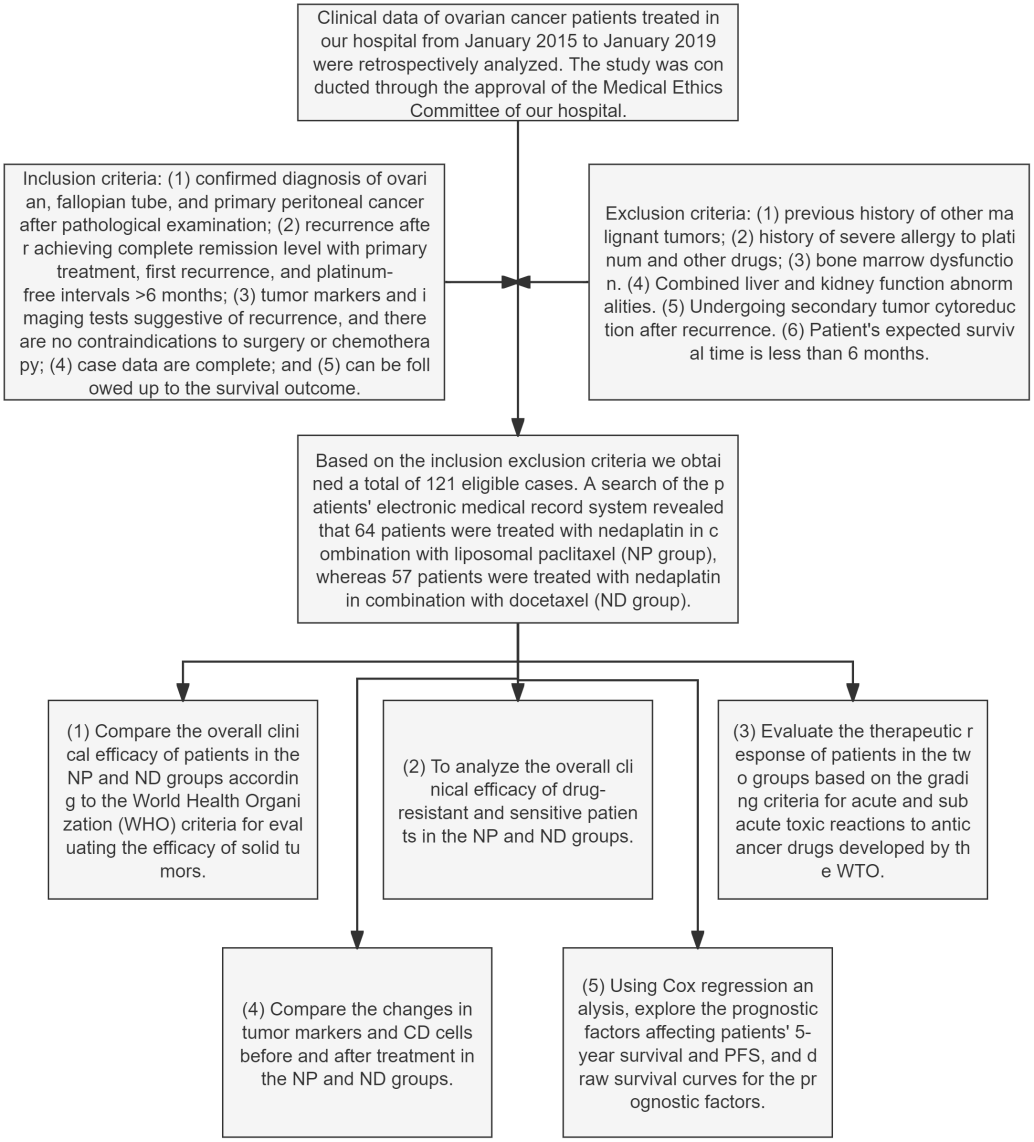
Flowchart for the research Paclitaxel liposome treatment (NP group), and Docetaxel treatment (ND group).

At the end of chemotherapy, the overall clinical outcomes of patients in the NP and ND groups were compared and analysed between drug-resistant and susceptible patients in the two groups, judged in the light of the World Health Organization (WHO) criteria for assessing the effectiveness on solid tumours (16). Changes in tumour markers and CD cells prior and post therapy were compared between the NP and ND groups, as assessed according to the WTO classification criteria for acute and subacute toxic reactions to anticancer drugs (17). Cox regression was conducted for analysing the prognostic factors affecting patients’ 5-year OS as well as PFS, and survival curves were plotted for the prognostic factors. The flow chart of this study is as follows (**Figure 1**).

### 2.7 Statistical analyses

This present study was conducted using GraphPad 8 software package to draw the required pictures. The distribution of the measured data was tested using the K-S test, and when the data were normally distributed data were tested using the t-test, intergroup comparisons were made using the independent samples t-test (for comparison of testing indicators between the pre-treatment and post-treatment NP and ND groups), and intragroup comparisons were made using the paired t-test (for comparison of testing indicators between the pre-treatment and post-treatment NP, ND groups), and were expressed in t. Non-normally distributed data were analysed by the rank sum test, and were expressed as Z. Count data were described through rate (%), using chi-square test, described by χ2, K-M survival curves were used to plot patients’ 2-year survival, and multifactorial Cox regression was performed for analysing the independent prognostic factors affecting 5-year OS and PFS in ROC patients. A variance inflation factor (VIF) was used to assess collinearity between each predictor variable in the model. According to conventional statistical criteria, VIF values greater than 10 are considered to indicate significant collinearity, while VIF values for all variables in our analysis are below 5, indicating that there is no significant collinearity problem in our model (**Supplementary Tables S1**, **S2**). P<0.05 implies a notable difference.

## 3 Results

### 3.1 Inter-group comparison of general clinical data

Inter-group comparison of the clinical characteristics revealed no statistically significant differences in age, FIGO staging, pathological type, initial surgical treatment, ECOG score, maximum diameter of recurrent lesions, number of recurrent lesions, response to platinum drugs, history of diabetes mellitus, and history of hypertension between the NP and ND groups (P>0.05, **Table 1**). The age, FIGO staging, pathological type, initial surgical treatment, ECOG score, maximum diameter of recurrent lesions, number of recurrent lesions, response to platinum drugs, history of diabetes mellitus and history of hypertension were not statistically different between the NP and ND groups (P>0.05, **Table 1**).

**Table 1:** Comparison of general clinical data between two patient groups.

Clinical characteristic		NP group(n=64)	ND group(n=57)	χ2/t	P
Age		54.00[44.75,66.00]	51.00[47.00,62.00]	0.719	0.473
FIGO staging					
	I-II	16	18	0.646	0.422
	III-IV	48	39		
Pathological type					
	Serous	38	30	0.557	0.455
	Other	26	27		
Initial surgical treatment					
	Yes	60	50	1.327	0.249
	No	4	7		
ECOG score					
	0–1	48	44	0.08	0.778
	≥2	16	13		
Maximum diameter of recurrent lesions					
	<4cm	42	29	2.704	0.1
	≥4cm	22	28		
Number of recurrent lesions					
	1	29	22	0.558	0.455
	>1	35	35		
Response to platinum drugs					
	Sensitivity	22	23	0.461	0.497
	Resistance	42	34		
History of diabetes mellitus					
	Present	14	16	0.621	0.431
	Absent	50	41		
History of hypertension					
	Present	16	11	0.565	0.452
	Absent	48	46		

International Federation of Gynecology and Obstetrics Staging (FIGO) staging, Eastern Cooperative Oncology Group Performance Status (ECOG) score.

Comparison of treatment efficacy between patients with resistance and sensitivity.

**Figure 2:**
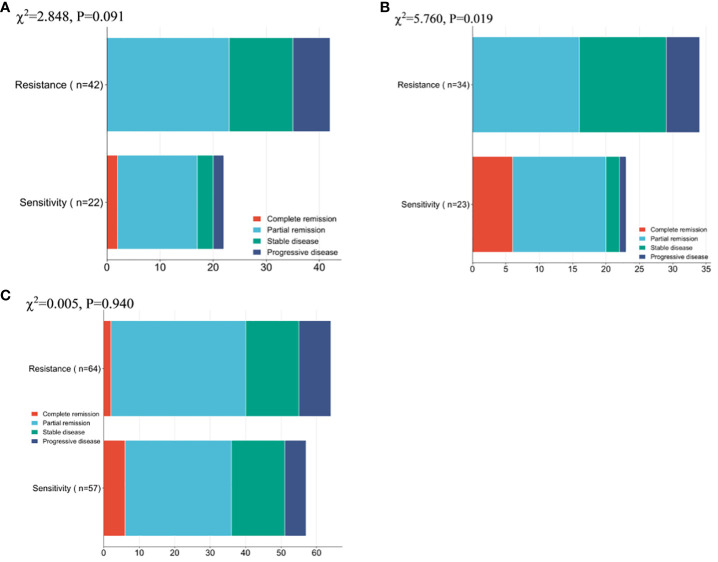
Patient Clinical Efficacy Assessment **(A)** Comparison of the overall number of remissions between drug-resistant and sensitive patients in the NP group. **(B)** Comparison of the overall number of remissions between drug-resistant patients and sensitive patients in the ND group. **(C)** Comparison of the overall number of patients in remission between patients in the NP group and patients in the ND group. Paclitaxel liposome treatment (NP group), and Docetaxel treatment (ND group).

Firstly, the remission of NP group patients was compared, and no notable difference was observed between the overall number of remissions of resistance patients and sensitivity patients (χ2 = 2.848,0.091), while a conspicuous difference was found between the overall number of remission of ND group resistance patients and sensitivity patients (χ2 = 5.760,0.016). Whereas there existed no notable inter-group difference regarding the overall remission rate (χ2 = 0.005,0.940, **Figure 2**).

### 3.2 Changes in tumour markers before and after treatment

**Figure 3:**
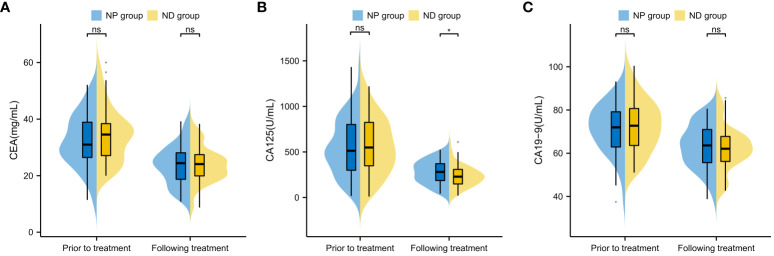
Comparison of Tumour Marker Changes Before and After Treatment in Patients **(A)** Inter-group Comparison of CEA Levels Before and After Treatment **(B)** Inter-group Comparison of CA125 Levels Before and After Treatment **(C)** Inter-group Comparison of CA19–9 Levels Before and After Treatment Note: Carcinoembryonic Antigen (CEA), Cancer Antigen 125 (CA125), Cancer Antigen 19–9 (CA19–9), Paclitaxel liposome treatment (NP group), and Docetaxel treatment (ND group).

The CEA, CA125 and CA19–9 levels were compared between the two groups prior and post treatment, and no conspicuous difference was found between the two groups in terms of CEA, CA125 and CA19–9 before treatment (P>0.05, **Figure 3**). The serum CEA, CA125 and CA19–9 in both groups decreased notably after therapy (P<0.05, **Figure 3**), but further comparison showed notably higher serum CA125 in NP group patients in contrast to ND group patients (P<0.05), whereas no notable difference existed between the two groups in CEA and CA19–9 (P>0.05, **Figure 3**). No notable inter-group difference was observed regarding CEA and CA19–9 after treatment (P>0.05, **Figure 3**).

### 3.3 Changes in immune function before and after treatment

**Figure 4:**
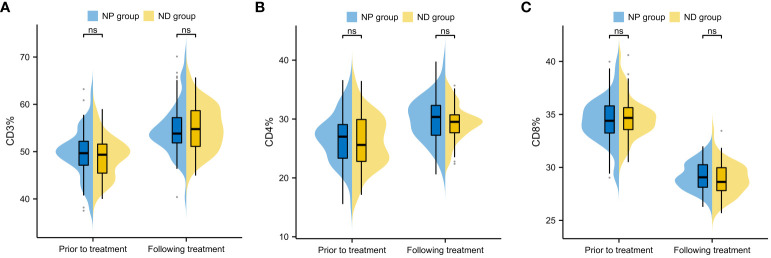
Comparison of changes in immune function indexes before and after treatment of patients **(A)** Inter-group comparison of CD3 level changes before and after treatment **(B)** Inter-group comparison of CD4 level changes before and after treatment. **(C)** Inter-group comparison of CD8 level changes before and after treatment. Cluster of Differentiation 3 (CD3), Cluster of Differentiation 4 (CD4), Cluster of Differentiation 8 (CD8), Paclitaxel liposome treatment (NP group), and Docetaxel treatment (ND group).

Inter-group comparison of CD3, CD4 and CD8 levels before and after treatment revealed no notable difference in CD3, CD4 and CD8 between the two groups before therapy (P>0.05, **Figure 4**). After therapy, the serum levels of CD3 and CD4 increased significantly, while CD8 decreased significantly (P<0.05, **Figure 4**), and further comparison showed no notable inter-group difference in the CD3, CD4 and CD8 levels between after treatment (P>0.05, **Figure 4**).

### 3.4 Statistical analysis of adverse reactions in two patient groups

The adverse reactions of the two groups revelled no notable differences between the two groups regarding leukopenia, thrombocytopenia, haemoglobin reduction, gastrointestinal reactions, muscle pain, hepatic impairment and renal impairment (P>0.05, **Table 2**).

**Table 2:** Account of adverse drug reactions.

Adverse reactions	NP group (n=64)			ND group (n=57)			χ2	P
	Grade 0	Grade I-II	Grade III-IV	Grade 0	Grade I-II	Grade III-IV		
Leukopenia	5	43	16	6	31	20	2.083	0.353
Thrombocytopenia	56	8	1	46	9	3	1.646	0.439
Anaemia	58	6	0	48	9	1	2.254	0.324
Gastrointestinal reactions	29	33	2	31	26	0	2.501	0.286
Myalgia	45	19	0	35	22	0	1.068	0.301
Hepatic impairment	35	29	0	33	23	1	1.351	0.509
Renal impairment	32	32	0	26	31	0	0.232	0.630

### 3.5 Survival analysis

**Figure 5:**
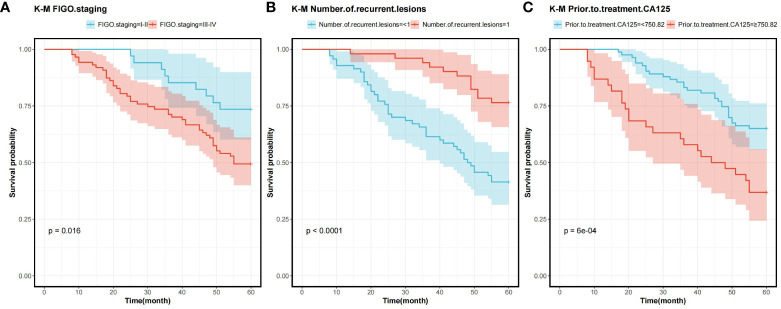
Survival curves for 5-year OS prognostic factors **(A)** 5-year OS curves comparing patients with different FIGO staging **(B)** 5-year OS curves comparing patients with different number of recurrent lesions **(C)** 5-year OS curves comparing patients with high and low CA125 expression. Overall survival rate (OS), International Federation of Gynecology and Obstetrics Staging(FIGO), and Cancer Antigen 125 (CA125).

**Figure 6:**
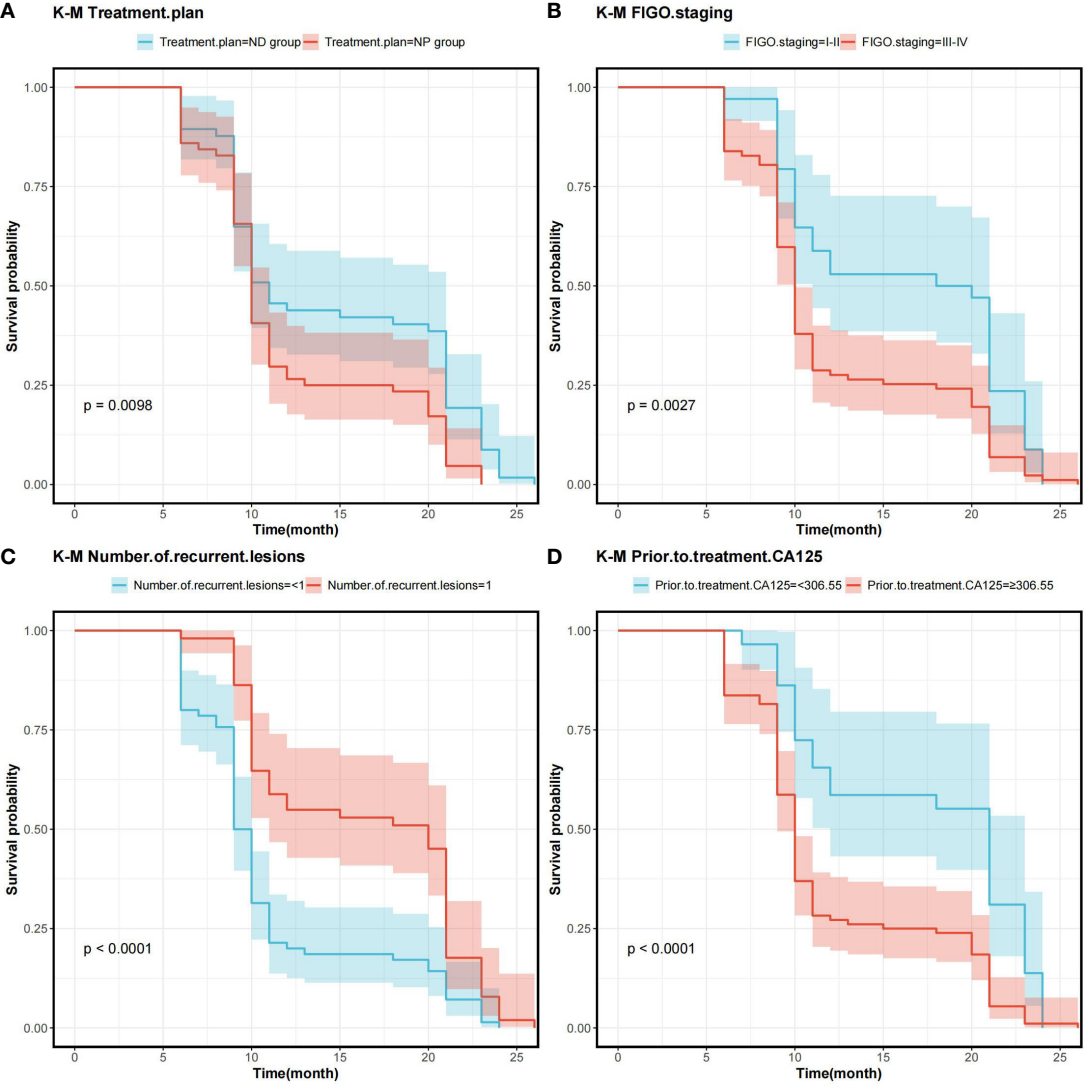
Survival curves on prognostic factors for PFS **(A)** PFS curves for comparing various treatment regimens in patients. **(B)** PFS curves based on different FIGO staging in patients. **(C)** PFS curves of patients with varying numbers of recurrent lesions. **(D)** PFS curves comparing patients with high and low CA125 expression Note: Progression-Free Survival (PFS), International Federation of Gynecology and Obstetrics Staging(FIGO), and Cancer Antigen 125 (CA125).

In order to determine the survival factors affecting ROC patients, we analysed patients’ five-year OS and PFS separately by Cox regression. Cox regression analysis of five-year survival identified FIGO staging (P=0.033, HR=0.456, 95%CI=0.222–0.938), number of recurrent lesions (P<0.001, HR=0.268, 95%CI=0.140–0.513) and pre-treatment CA125 (P=0.005, HR=1.001, 95%CI=1.000–1.002) as independent prognostic factors for 5-year OS in ROC patients (**Table 3**, **Figure 5**). While PFS Cox regression analysis identified treatment regimen (P=0.004, HR=1.759, 95%CI=1.196),FIGO staging (P=0.007, HR=0.568, 95%CI=0.376–0.858), number of recurrent lesions (P<0.001, HR=0.416, 95%CI=0.285–0.608) and pre-treatment CA125 (P=0.002, HR=1.001, 95%CI=1.000–1.002) as the independent prognostic factors for PFS of ROC patients (**Table 4**, **Figure 6**).

**Table 3:** Five-year OS in patients with recurrent ovarian cancer Cox regression analysis.

	Univariate Cox regression				Multivariate Cox regression			
	P	HR	Lower	Upper	P	HR	Lower	Upper
Treatment regimen	0.932	0.977	0.570	1.675				
Age	0.196	1.434	0.830	2.476				
FIGO staging	0.020	0.427	0.208	0.875	0.033	0.456	0.222	0.938
Pathological type	0.673	1.126	0.649	1.953				
Initial surgical treatment	0.740	1.189	0.429	3.294				
ECOG score	0.390	1.339	0.689	2.601				
Maximum diameter of recurrent lesions	0.860	0.952	0.553	1.640				
Number of recurrent lesions	0.000	0.285	0.149	0.543	<0.001	0.268	0.140	0.513
Response to platinum drugs	0.731	1.101	0.635	1.910				
History of diabetes mellitus	0.622	1.166	0.633	2.147				
History of hypertension	0.082	0.513	0.242	1.088				
Pre-treatment CA125	0.007	1.001	1.000	1.002	0.005	1.001	1.000	1.002
Pre-treatment CA19–9	0.890	1.002	0.979	1.025				
Pre-treatment CEA	0.380	0.987	0.958	1.016				
Pre-treatment CD3	0.686	1.013	0.953	1.076				
Pre-treatment CD4	0.816	1.007	0.948	1.070				
Pre-treatment CD8	0.762	0.980	0.861	1.116				

International Federation of Gynecology and Obstetrics Staging(FIGO) staging, Eastern Cooperative Oncology Group Performance Status (ECOG) score, Carcinoembryonic Antigen (CEA), Cancer Antigen 125 (CA125), and Cancer Antigen 19–9 (CA19–9), Cluster of Differentiation 3 (CD3), Cluster of Differentiation 4 (CD4), and Cluster of Differentiation 8 (CD8).

**Table 4:** Five-year PFS in patients with recurrent ovarian cancer Cox regression analysis.

	Univariate Cox regression				Multivariate Cox regression			
	P	HR	Lower	Upper	P	HR	Lower	Upper
Treatment regimen	0.009	1.660	1.136	2.425	0.004	1.759	1.196	2.588
Age	0.689	1.081	0.738	1.584				
FIGO staging	0.006	0.569	0.380	0.852	0.007	0.568	0.376	0.858
Pathological type	0.531	1.123	0.781	1.614				
Initial surgical treatment	0.451	1.271	0.681	2.373				
ECOG score	0.660	1.101	0.718	1.687				
Maximum diameter of recurrent lesions	0.171	1.294	0.895	1.871				
Number of recurrent lesions	0.000	0.477	0.329	0.690	0.000	0.416	0.285	0.608
Response to platinum drugs	0.894	0.975	0.670	1.418				
History of diabetes mellitus	0.617	1.112	0.733	1.686				
History of hypertension	0.203	0.756	0.491	1.163				
Pre-treatment CA125	0.005	1.001	1.000	1.001	0.002	1.001	1.000	1.002
Pre-treatment CA19–9	0.197	0.990	0.975	1.005				
Pre-treatment CEA	0.298	0.989	0.970	1.009				
Pre-treatment CD3	0.723	1.008	0.966	1.051				
Pre-treatment CD4	0.793	1.005	0.967	1.045				
Pre-treatment CD8	0.829	1.010	0.922	1.106				

International Federation of Gynecology and Obstetrics Staging (FIGO) staging, Eastern Cooperative Oncology Group Performance Status (ECOG) score, Carcinoembryonic Antigen (CEA), Cancer Antigen 125 (CA125), and Cancer Antigen 19–9 (CA19–9), Cluster of Differentiation 3 (CD3), Cluster of Differentiation 4 (CD4), and Cluster of Differentiation 8 (CD8).

## 4 Discussion

Even after standardised treatment, the overall survival of OC patients is still unsatisfactory, mainly due to the high recurrence rate of OC (18, 19). For ROC patients, the main goals of treatment include prolonging survival and lifting quality of life. Therefore, an in-depth study of the factors affecting ROC and a comparison of the effects of different treatments are of great clinical significance to increase the PFS from the first recurrence to the next progression of the disease and to improve the overall survival after recurrence.

In the study, we found no notable difference in the number of overall remissions between patients with resistance and sensitivity in the NP group, while the number of overall remissions in patients with sensitivity in the ND group was higher in contrast to patients with resistance, and there was no inter-group difference in the statistics of toxic side effects. It is suggested that aqupla in combination with docetaxel is more suitable for the therapy of platinum-sensitive ROC and does not increase the side effects in patients. We believe that this is due to the fact that docetaxel, as a microtubule stabiliser, inhibits cell division by preventing the depolymerisation of microtubules, leading to cancer cell death (20). Although its mechanism is similar to that of paclitaxel, docetaxel has a different chemical structure and exhibits stronger inhibitory effects on resistant tumour cells. This is particularly true in patients exhibiting resistance to platinum-based drugs or paclitaxel, and is the reason why docetaxel is more effective in the therapy of patients with aqupla-sensitive ROC.

CA125, as one membrane-associated protein, is extensively adopted in the diagnosis, therapy monitoring and recurrence monitoring of OC. Although it is not a marker specific to OC, it plays an important role in diagnostic assistance, assessment of treatment efficacy, monitoring of disease recurrence, and prognostic assessment (21, 22). For example, Liu et al. (23) suggested that increased serum levels of CA125 are one biomarker that can be adopted for modifying the prognosis of OC as determined by BRCA mutations and family history. Additionally, Gong et al. (24) implied that elevated expression of CA125 is strongly bound up with the condition of OC, and when its expression exceeds 175.243 kU/L, it suggests that patients with OC have a high risk of unfavourable prognosis, and should be intervened in early stage to prevent the recurrence or metastasis of OC. We found that the changes of tumour markers and CD cells were positive in both groups through treatment, but interestingly, except for CA125, the rest of the indicators were not notably different between the two groups after treatment. In the ND group, CA125 was lower than that of NP patients after treatment, suggesting that the aqupla combined with docetaxel regimen may be more effective in reducing the tumour load. Therefore, in the treatment and management of OC, changes in the CA125 level can reflect the response to treatment, in which a decrease in the level usually indicates that the treatment is effective, while an increase in the level can indicate progression or recurrence of the disease, which should be intervened and prevented at an early stage.

Screening for prognostic factors in ROC patients is essential for identifying key variables, and by analysing these factors, physicians are able to optimise treatment strategies, improve treatment outcomes and patients’ quality of life (25, 26), and provide important guidance in the development of novel therapeutic approaches and the formulation of effective follow-up plans.

At the end of the study we used Cox regression to screen the factors affecting 5-year OS and PFS of patients. Our results showed that FIGO staging, number of recurrent lesions and pretreatment CA125 were independent prognostic factors for 5-year OS and PFS in ROC patients. And interestingly we found that ND regimen prolonged PFS in ROC patients. FIGO staging ≥ III-IV means that the cancer is more advanced and has spread to the peritoneum or lymph nodes. In this case, the cancer is not only more difficult to treat, but also increases the chance of recurrence. **Table 1** also shows a higher number of patients with stage III-IV in contrast to that of stage I-II, which means that the higher the staging, the worse the prognosis of the patients usually is. For example, FIGO III-IV was identified as one independent prognostic factor for OS and PFS in OC patients in research by Bai et al. (27). In addition, a SEER database –based study proposed FIGO staging as an independent prognostic factor for malignant germ cell tumours of the ovary (28). In addition, a study by Shibuya (29) et al. similarly suggested that an increase in clinical stage leads to a poorer postoperative prognosis in patients with OC, resulting in a decrease in the overall survival of the patients. An increase in the number of recurrent lesions reflects the extensive and heterogeneous nature of the tumour and is indicative of a greater capacity for tumour survival and spread. Multiple recurrent lesions imply that the tumour is resistant to prior treatment and more difficult to control with local therapy, leading to a poorer prognosis. In a multicentre study, an analysis of prognostic factors in 670 patients with recurrent epithelial OC revealed a notably worse prognosis in patients with ≥3 recurrent sites (30). Also in a study by Fan et al. (31), it was found that patients with 1 recurrent lesion had significantly longer median survival and OS compared to patients with recurrent epithelial OC with more than 1 lesion.

Additionally, research by Zang et al. (32) suggested that the extent of recurrent disease (single or multiple) is critical in determining the prognosis of patients with ovarian tumours of malignant potential. CA125 is one crucial biomarker for OC, and its high level is often linked to high tumour load and disease activity. The association of CA125 with OC prognosis has been reported in several articles. For example, Fleming et al. (33) suggested that continuous CA125 monitoring for early detection of recurrence might improve the optimal rate of secondary cytoreduction and potentially impact the overall survival of ROC patients. Another report showed (34) that the therapy effect of platinum-refractory/resistance ROC could be predicted by the reduction of CA125 levels after 2 courses of treatment. The ND regimen prolongs PFS in ROC patients, mainly because of the unique mechanism of action of docetaxel and its synergistic effect with aqupla. Docetaxel, as a microtubule stabiliser, is able to inhibit cancer cell division by hindering microtubule depolymerisation, a mechanism that may show enhanced activity in cancer cells resistant to conventional therapy (35–37). When combined with aqupla, a platinum drug capable of forming DNA cross-links, the two drugs act at different phases of the cell cycle, generating a powerful synergistic effect that enhances anti-tumour activity and thus prolongs PFS more effectively.

## 5 Study limitations

In the present study, we faced several key limitations in examining the effects of NP versus ND regimens on survival and biomarkers in ROC patients. First, the study in retrospective design probably has been affected by selection bias and information bias. Additionally, the number of samples in the study was limited, comprising only 121 patients, and this small sample size may have affected the efficacy of the statistical analyses. Finally, as a single-centre study, extrapolation of the results may be limited because the population and treatment setting covered by the study might not be reflective of other regions or countries. We hope future research will need to validate and extend our findings using a larger, multicentre and randomised controlled design to verify the conclusions.

## 6 Future research directions

Given these limitations, future studies should aim to validate and expand upon our results. A larger, multicentre approach is crucial to increase sample size and variability, providing a more comprehensive analysis. Additionally, adopting a randomized controlled design can offer more robust evidence by reducing biases and confounding factors. Such research would be invaluable in verifying our conclusions and enhancing the external validity of these findings, thereby enabling better-informed clinical decisions regarding NP and ND regimens in ROC patients.

## 7 Conclusion

In ROC patients, particularly platinum-sensitive patients, the aqupla in combination with docetaxel regimen provided an improved survival benefit with a comparable safety profile, underscoring the importance of individualised treatment strategies.

## Data availability statement

The original contributions presented in the study are included in the article/**Supplementary Material**. Further inquiries can be directed to the corresponding author.

## Ethics statement

The studies involving humans were approved by ethics committee of Women’s and Children’s Hospital of Ningbo University. The studies were conducted in accordance with the local legislation and institutional requirements. The participants provided their written informed consent to participate in this study.

## Author contributions

JY: Conceptualization, Data curation, Methodology, Writing – original draft. MZ: Formal analysis, Software, Validation, Writing – original draft. YZ: Formal analysis, Methodology, Writing – original draft. LZ: Formal analysis, Investigation, Writing – original draft. QW: Formal analysis, Supervision, Writing – review & editing.
