# Supplementary material for: Combined aqupla, paclitaxel liposome, and docetaxel treatment: survival and biomarker outcomes in recurrent ovarian cancer patients
Source: Front Oncol. 2024 Jun 17;14:1422117. doi: 10.3389/fonc.2024.1422117 (PMC11215079; doi:10.3389/fonc.2024.1422117)
Supplement: Supplementary file 1 [file Table_1.docx]

Table S1 Total Survival Cox regression Multivariate Analysis of VIF

| Variable | Type | VIF |
| --- | --- | --- |
| FIGO staging | Level Variables |  |
| III-IV |  | Reference |
| I-II |  | 1.0055 |
| Number of recurrent lesions | Level Variables |  |
| =1 |  | Reference |
| >1 |  | 1.0044 |
| Pre-treatment CA125 | Numeric variables | 1.0096 |

Table S2 PFS Cox regression multivariate analysis of VIF

| Variable | Type | VIF |
| --- | --- | --- |
| Treatment regimen | Level Variables |  |
| ND |  | Reference |
| NP |  | 1.0056 |
| FIGO staging | Level Variables |  |
| III-IV |  | Reference |
| I-II |  | 1.0225 |
| Number of recurrent lesions | Level Variables |  |
| =1 |  | Reference |
| >1 |  | 1.0234 |
| Pre-treatment CA125 | Numeric variables | 1.0262 |
